# Bidirectional Linear Motion by Travelling Waves on Legged Piezoelectric Microfabricated Plates

**DOI:** 10.3390/mi11050517

**Published:** 2020-05-20

**Authors:** Víctor Ruiz-Díez, Jorge Hernando-García, Javier Toledo, Abdallah Ababneh, Helmut Seidel, José Luis Sánchez-Rojas

**Affiliations:** 1 Microsystems, Actuators and Sensors Group, Universidad de Castilla-La Mancha, E-13071 Ciudad Real, Spain; jorge.hernando@uclm.es (J.H.-G.); javier.toledo@uclm.es (J.T.); joseluis.saldavero@uclm.es (J.L.S.-R.); 2 Electronic Engineering Department, Hijjawi Faculty for Engineering Technology, Yarmouk University, Irbid 21163, Jordan; a.ababneh@yu.edu.jo; 3 Chair of Micromechanics, Microfluidics/Microactuators, Faculty of Natural Sciences and Technology, Saarland University, 66123 Saarbrücken, Germany; seidel@lmm.uni-saarland.de

**Keywords:** travelling wave, bidirectional linear motion, conveyor, piezoelectric, AlN, MEMS

## Abstract

This paper reports the design, fabrication and performance of MEMS-based piezoelectric bidirectional conveyors featuring 3D printed legs, driven by linear travelling waves (TW). The structures consisted of an aluminium–nitride (AlN) piezoelectric film on top of millimetre-sized rectangular thin silicon bridges and two electrode patches. The position and size of the patches were analytically optimised for TW generation in three frequency ranges: 19, 112 and 420 kHz, by the proper combination of two contiguous flexural modes. After fabrication, the generated TW were characterized by means of Laser–Doppler vibrometry to obtain the relevant tables of merit, such as the standing wave ratio and the average amplitude. The experimental results agreed with the simulation, showing the generation of a TW with an amplitude as high as 6 nm/V and a standing wave ratio as low as 1.46 for a device working at 19.3 kHz. The applicability of the fabricated linear actuator device as a conveyor was investigated. Its kinetic performance was studied with sliders of different mass, being able to carry a 35 mg silicon slider, 18 times its weight, with 6 V of continuous sinusoidal excitation and a speed of 0.65 mm/s. A lighter slider, weighting only 3 mg, reached a mean speed of 1.7 mm/s at 6 V. In addition, by applying a burst sinusoidal excitation comprising 10 cycles, the TW generated in the bridge surface was able to move a 23 mg slider in discrete steps of 70 nm, in both directions, which is a promising result for a TW piezoelectric actuator of this size.

## 1. Introduction

The field of micro-electro-mechanical systems (MEMS) comprises a great variety of sensors and actuators that represent a rising platform for several applications, such as telecommunications, chemistry, biosensors or automotive engineering [1,2]. The benefits associated with miniaturization allowed for improved performance, compact size, low power consumption, array configurations and low manufacturing costs in MEMS sensors. On the contrary, the miniaturization of actuators is still a challenge [3], especially in applications that require large displacements, high energy efficiency or output forces. In this regard, piezoelectric ultrasonic motors (USM) arose as a solution to obtaining a long motion range, high torque, quick response, high power to weight ratio and high efficiency in comparison to electrostatic, magnetic, and thermal approaches [4,5,6].

USM are devices based on a rotor or slider pressed against a stator surface, where elastic vibrations induce rotational or linear movement by friction between surfaces [7,8,9]. USM can be classified, according to the vibration nature, as standing-wave (SW) or travelling-wave (TW), or to the principal axis of movement, as rotatory or linear. Early commercial motors were implemented using circular TW-based stators transmitting its energy to a rotor [10,11], with the capability of bidirectional movement. SW-based linear motors were also reported, with the requirement of legs to induce the movement of the rotor [12], as well as by the proper mixing of two standing waves at the same frequency [13,14]. Linear USM can also be implemented by just combining two contiguous bending modes, without the degeneration requirement, which results in less restrictions on the design of the device [15]. Different reports already demonstrated centimetre-sized robotic devices that move on solid surfaces with this approach [16,17,18,19], or even in liquid media [20].

Despite the advantages of USM for linear positioning stages, locomotion or conveyance, scaling down to the millimetre range remains a challenge [21]. The work by Kurosawa [22] is a reference to mention for linear motors at these scales, demonstrating frictional driven motion by surface acoustic waves (SAW) on a LiNbO_3_-actuated stator and a 4 × 4 mm^2^ slider. Due to the low amplitude of the generated waves, preloads and voltages as high as 30 N and 80 V, respectively, were required, though speeds of more than 1 m/s, with minimum steps of 25 nm, were obtained. 

In pursuit of miniaturization, the monolithic fabrication based on silicon micromachining was successfully applied to the effective size reduction of such positional devices. In Kladitis et al.’s work, [23] a 10 × 10 mm^2^ robot, based on an array of electrothermally actuated and manually attached silicon legs, was able to move a 3 mg slider at a speed of 8 µm/s with a maximum payload of 40 mg in just one direction. Ebefors et al.’s work [24] relied on electrothermal actuation to develop a hybrid silicon–polyimide walker, capable of reaching speeds of 6 mm/s at 18 V. In a more recent work, a 9 × 9 mm^2^ monolithic conveyor based on tilted air jets demonstrated the ability to move 2 mg objects within the device plane [25]. However, positional precision was not reported for this complex system that required four high-pressure-controlled electrovalves.

The use of piezoelectric films for an all-electrical actuation scheme, integrated into the monolithic fabrication process, allowed for a further step into the miniaturisation of efficient motors. In this regard, TW-based bidirectional rotatory motion in the millimetre scale was demonstrated using thin-film PZT on silicon rotors with teeth by two degenerate orthogonal modes [26]. The device was able to reach rotational speeds above 1500 rpm at 5 V, but tight design requirements made it difficult to scale down beyond the millimetre [27]. Other monolithic devices for conveyance at this scale, but not relying on TW actuation, can be found in the literature. Tellers et al. [28] proposed a different approach, where an array of PZT-actuated micro-hammers worked as a linear conveyor, capable of transporting 2 mg masses at 1 mm/s with 5 V excitation and positional errors as low as 2%, without speed control. Towards the goal of miniaturization, recent research [29] proved the feasibility of linear TW generation in AlN-actuated devices in the mili-to-micro scale. However, the practical motion of objects on top of the device was not implemented due to difficulties associated with both the static deformation produced by built-in stress and the fragility of the suspended structure. A summary of the state of the art of miniature linear motors can be found in Table 1.

In this work, TW-based bidirectional linear motors were fabricated with a micromachined silicon plate and 3D-printed legs. The MEMS devices were actuated thanks to an integrated AlN piezoelectric film. An analytical approach was used to calculate the optimal electrode layout for an efficient TW generation, while, experimentally, laser Doppler vibrometry allowed for the assessment of the TW. The movement of the surface of the silicon plate was transferred to a slider through an array of 3D-printed legs attached to its surface, allowing a controlled contact between stator and slider and the amplification of the elliptical movement caused by the TW. The conveyance of different sliders was demonstrated and the kinetic and positioning characteristics of the motor were characterised.

## 2. Device Design

**Figure 1:**
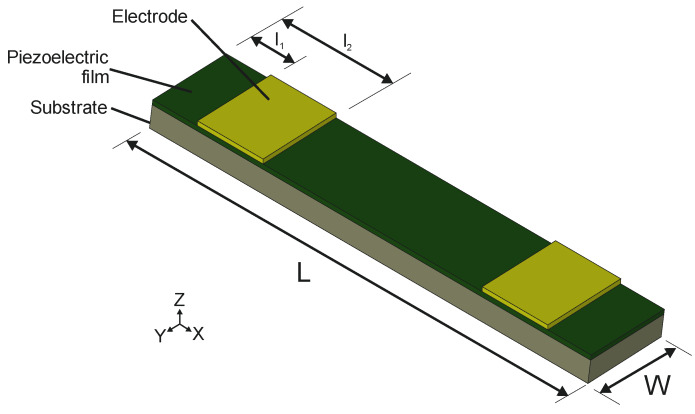
Schematic diagram of the device design. A bridge structure of length L and width W consisting of a silicon substrate with thickness $t_{s}$, covered by an AlN piezoelectric layer of thickness tp. Two symmetrically disposed metallic electrodes were placed closed to the edges, starting at a distance $l_{1}$ and ending at a distance $l_{2}$.

Here, we focus on the TW generation in piezoelectrically actuated bridges with a length L=10 mm and a width W=2 mm. The structure consisted of a silicon substrate with a thickness $t_{s}=30\;\mu m$, an aluminium–nitride (AlN) piezoelectric film with a thickness $t_{p}=1\;\mu m$ and two 500 nm thick Gold (Au) electrode patches on top, that were neglected in the mechanical analysis. AlN was preferred as piezoelectric material due to its demonstrated compatibility with the standard CMOS process, low losses and an acceptable piezoelectric coefficient [30]. Figure 1 depicts the layout, while the electromechanical properties assumed for the device design are summarised in Table 2.

**Figure 2:**
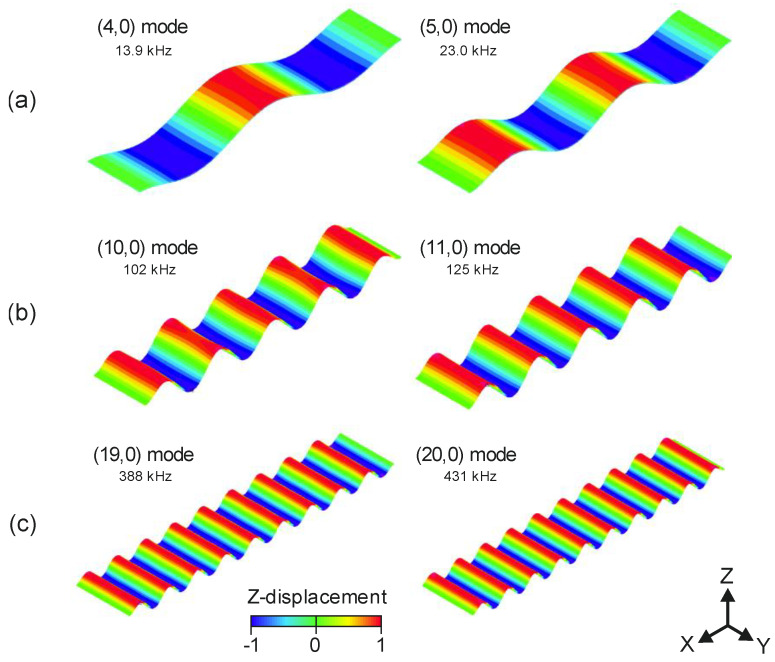
Mode shapes involved in the travelling waves (TW) generation obtained from the finite element method (FEM) analysis. (**a**) Modes (4,0) and (5,0) in the low frequency (LF) range, (**b**) modes (10,0) and (11,0) in the intermediate frequency (IF) range and (**c**) modes (19,0) and (20,0) in the high frequency (HF) range. Resonant frequency is also included. Colour bar represents the normalised modal displacement in the *Z*-axis.

The generation of linear TW in beams by the combination of two flexural vibration modes was already reported, using two symmetrically placed piezoelectric patches [16,18,29,34]. Here, we followed the same approach. In the following sections, the flexural resonant modes were named using two numbers, based on Leissa nomenclature for bending modes [35]. Each number was used to designate the amount of nodal lines along the two orthogonal directions on the plane: the first one indicated the number of nodal lines in the longitudinal direction, while the second one indicated the number of nodal lines in the transverse direction. Three frequency ranges were studied: the low frequency (LF) range for the combination of modes (4,0) and (5,0); the intermediate frequency (IF) range with modes (10,0) and (11,0); and the high frequency (HF) range with modes (19,0) and (20,0). Figure 2 shows the mode shapes and the corresponding resonant frequencies obtained from a finite element method (FEM) modal analysis. The model from Figure 1 was implemented in the FEM software ADINA, including the silicon substrate and the AlN piezoelectric film in a bridge structure, clamped at both ends. A damping ratio of 0.001 was assumed in the design phase, as measured for the flexural modes of similar AlN-based devices working in air [36].

Our first objective was to determine the size and the position of the patches that optimized the performance of the devices. Two figures of merit were used to assess the performance: (i) Standing Wave Ratio (SWR), defined as the ratio of the maximum to the minimum value of the TW envelope along the length of the plate, which is related to the quality of the TW; the closer to 1, the better the TW, and (ii) the average displacement of the TW envelope, named <TW>, which is associated with the energy of the wave.

Following the same procedure as in Hernando-García et al.’s research [18] and according to basic mode superposition [37], the vertical displacement v(x,t) at any time and position along the length of the structure can be expressed by

(1) $$v\left(x,t\right)=\overset{\infty }{\underset{i=1}{\sum }}\varphi_{i}\left(x\right)T_{i}\left(t\right)$$

where $\varphi_{i}$ are the shapes of the normalized flexural modes and $T_{i}$ are the time-dependent modal coefficients. This time-dependent modal factor can be Fourier transformed to the following expression for each of the patches with an actuation voltage $Ve^{j\left(\omega t+\phi \right)}$ in the complex domain [34,38]

(2) $$T_{i}\left(\omega \right)=-\frac{Y_{p}d_{31}\left(\frac{W_{s}}{2}\right)t_{p}\left(t_{p}+t_{s}-2z_{n}\right)\left(\varphi^{\prime }\left(l_{1}\right)-\varphi^{\prime }\left(l_{2}\right)\right)\;}{t_{p}\left(\omega_{i}^{2}-\omega^{2}+2j\xi_{i}\omega \omega_{i}\right)}Ve^{j\phi }$$

where $Y_{p}$ is the Young modulus of the piezoelectric film, $d_{31}$ is the piezoelectric coefficient, $z_{n}$ is the neutral axis of the laminate structure, $\varphi^{\prime }$ is the first spatial derivative of the modal shape, $l_{1}$ and $l_{2}$ are the initial and final positions of the patch, respectively, ω is the frequency of actuation, $\omega_{i}$ and $\xi_{i}$ are the resonance frequency and damping ratio of mode i. The TW envelope is taken as the magnitude of v(x,ω) for each position x at a given frequency.

In order to find the optimal patch, an optimization method was applied to Equation (2) for the three ranges of frequency considered. Following the approach of previous publications for the effective generation of a TW [18,34], the driving frequency was fixed to the mid-frequency between the resonant frequencies of the two modes under consideration, with a fixed phase shift of 90° between the sinusoidal signals applied to the patches. As shown in Hernando-García et al.’s research [18], if the driving frequency approximates the resonant frequency of either of the modes, the mean average amplitude of the TW increases (due to the resonance amplification) but the quality of the generated TW rapidly decreases (SWR increases). Distances $l_{1}$ and $l_{2}$, corresponding to the initial and the final positions of the patch, respectively, were varied in steps of 10 µm. For each combination of $l_{1}$ and $l_{2}$, SWR and <TW> were evaluated within a window at the centre of the structure, covering 50% of the total length, in order to avoid the effect of the boundary conditions. The optimization was carried out by selecting those patches that maximize the TW amplitude, <TW>, while ensuring an acceptable SWR. In our case, a maximum SWR of 1.4 was chosen.

**Figure 3:**
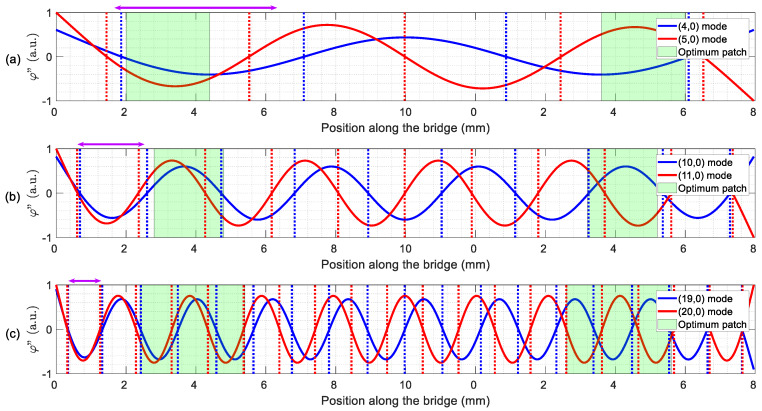
Second derivatives $\varphi^{\prime \prime }$ (solid lines) of the modes involved in the TW generation in the (**a**) low frequency (LF) range, (**b**) intermediate frequency (IF) range and (**c**) high frequency (HF) range. The positions of the zeros (dotted vertical lines) and optimum patch design (green area) are also indicated. A double-headed arrow indicates the patch based on the zeros of the second derivative.

Figure 3 shows the deduced optimal patches (green areas) with the second derivative of the modal shapes involved in the TW generation, as well as dotted vertical lines at their zeros. It is already well established in the literature [38,39] that the optimal actuation of each individual mode takes place with a top electrode covering the space between these zeros for that particular mode. A recent article [18] showed how the maximum <TW>, without accounting for SWR, was reached for patches covering approximately the distance between the midpoint of these zeros for the two modes, in free–free structures. In Figure 3, we indicate the patch inferred from this approximation by a double-headed arrow, from the midpoint between the first zeros to the midpoint of the second zeros. In what follows, we present the simulation results for both type of patches for comparison purposes.

**Figure 4:**
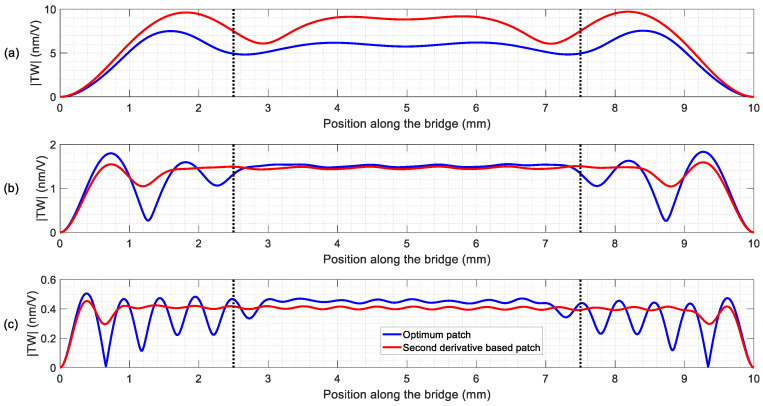
Resultant envelopes from the simulations with optimal patches and patches deduced from the approach based on the zeros of the second derivative in the (**a**) LF range, (**b**) IF range and (**c**) HF range. The dotted vertical lines indicate a centred 50% of the length.

Figure 4 compares the envelope of the TW for the calculated optimal patches and for the patches deduced from the approximation of the zeros of the second derivative. Table 3 was obtained after evaluating those envelopes. As can be seen, the systematic procedure provided optimal patches that allowed for the generation of good quality TW, with SWR below the 1.4 threshold chosen. Besides, the average amplitudes were higher than those of the second derivative-based patches, except in the LF range. There, the optimal patch improved the SWR at the cost of the average amplitude.

Patches based on the zeros of the second derivative provided good but inconsistent results when compared to those from the systematic procedure: higher <TW> at the LF range but worse SWR, while better SWR and lower <TW> at the IF and HF ranges. Unlike reference [18], there is not an obvious correlation between the optimal patches and the second derivative zeros approach. This might be related to the fact that, in reference [18], the value of $l_{1}$ was fixed to 0, i.e., the patches started at the edge of the devices, while here the value of $l_{1}$ was a new variable to consider. As a result, the devices for this work were fabricated according to the calculated optimal patches, although the patches deduced from the zeros of the second derivative might be a reliable option when an easy implementation is considered.

Once the TW generation was achieved, the next step was to ensure the contact of the vibrating structure with the slider to be transported. This is usually attained by a preloading [22]. In our case, such a procedure was discarded due to the suspended nature of the devices and the micrometric thickness. Besides, we must also deal with built-in stresses that result in a static deformation of hundreds of nm [29]. In order to circumvent such difficulties, the inclusion of millimetre-long legs was carried out, following the procedure reported already for mobile miniature robots [18]. According to the former reference, in order to ensure an elliptical trajectory at the tip of the legs, TW envelope should be ideally constant. By returning to Figure 4, we noticed that the positions to consider were those located at the central plateau of the TW envelope.

## 3. Materials and Methods

Monolithic microfabrication techniques were used to implement rectangular microplates according to the geometry and materials previously described, clamped at both sides. For all the devices, the layer structure was as follows: a 30-μm thick, p-doped (100), silicon plate served as the bottom electrode, which was covered with a 1-μm thick AlN piezoelectric film synthesized in a reactive sputter process from an aluminium target in pure nitrogen atmosphere. As the top electrode, 500-nm thick Au electrodes were deposited. A complete description of the fabrication process can be found in Ababneh et al.’s research [36].

**Figure 5:**
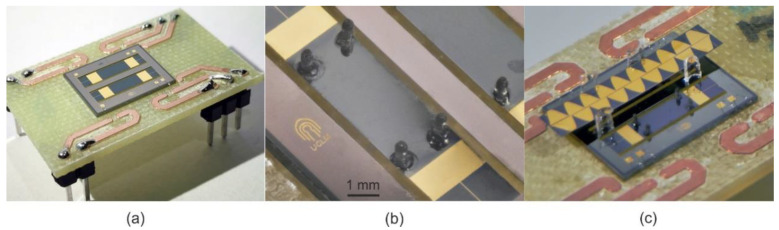
Photographs of the fabricated devices. (**a**) Silicon dice containing two HF designs, wire bonded to a printed circuit board (PCB), (**b**) detail of the legs attached and (**c**) the experimental setup, with a gold-patterned slider on top of a legged TW motor, and constrained to a lane by four glass pieces.

Three different top electrode layouts were implemented, considering the optimum patches obtained in the previous section (see Figure 3) for each frequency considered. The devices will be designated as LF, IF and HF for the low, intermediate and high driving frequencies, respectively. Dices with two different devices were glued and wire-bonded to a printed circuit board (PCB) to facilitate the electrical access (Figure 5a).

Cylindrical legs with a length of 750 µm and diameter of 300 µm were designed so that their fundamental resonant frequency was higher than the TW driving frequency, in order to avoid mechanical coupling effects between plate and legs. Longer legs would lead to a higher amplification of the displacement of the leg tip parallel to the plate, as reported in Hernando-García et al.’s research [18], but it would also lower the frequency of the leg’s modal resonances, so it could not be considered a rigid body. Due to the fabrication technology tolerances, the leg diameter was fixed to a minimum of 300 µm. These legs were manufactured using a B9 Core 530 DLP 3D printer (B9Creations, Rapid City, SD, USA), using proprietary Black Resin (density of $\rho =1150\;\mathrm{kg}/m^{3}$, Young modulus of E=1.75 GPa). This material allowed a soft contact while assuring an acceptable wear rate, in combination with the rapid prototyping of the 3D printing technology. Four of them were glued on the bridge surface using a cyanoacrylate-based adhesive (see Figure 5b). Their position was determined by the measured TW envelope, being symmetrically placed on the central plateau for better stability of the sliders. The fabricated devices were optically and electrically characterized prior and after leg attachment.

The electrical performance of the AlN-actuated devices was analysed by recording the impedance spectrum of the different modes of vibration using an all-electrical actuation/detection scheme on one of the two top electrodes. For this purpose, a 4294A Agilent impedance analyser (Agilent Technologies, Santa Clara, CA, USA) was used. The obtained impedance spectra was then fitted to a modified Butterworth–Van-Dyke equivalent circuit model, in order to obtain the equivalent electrical parameters of motion [40]. The resonant frequency of each mode and other figures of merit, such as the quality factor and the motional resistance, could be derived from these electrical parameters.

For the optical characterization, a scanning laser Doppler vibrometer (Polytec MSV 400, Polytec GmbH, Waldbronn, Germany) was used. This instrument provides a laser spot, which can scan a grid of points on the top device surface, to measure the out-of-plane component of either the velocity or the displacement. The characterization of both the modes of vibration and the generated TW was accomplished with this technique. The TW was generated by applying sinusoidal excitations on both electrode patches, with a phase difference of 90° and a driving frequency corresponding to the intermediate frequency between the two modes involved, while recording the out-of-plane displacement of the whole device surface. The figures of merit of the TW generation were calculated in a window located at the centre of the device, covering 50% of the length, as in the device design section.

Finally, for the kinetic characterization, a Tektronix AFG 3000 series arbitrary waveform generator (AWG, Tektronix U.K Limited, Oldbury, UK) was used to generate the required waveforms to be applied to each of the electrode patches. This AWG was controlled with LabVIEW software, allowing a fast switch between different wave parameters in the experiments. In the experimental setup, the PCB containing a pair of such devices (see Figure 5c) was placed on a levelled platform—in order to avoid an uneven movement of the slider—under the microscope camera. The orthogonal movement of the sliders was restricted to a rectilinear lane with the help of glass pieces. The movement of the slider was optically recorded by a microscope camera and the videos were processed by an optical character recognition (OCR) code programmed in Matlab, in order to obtain the slider positions versus time. To facilitate the image processing, a sinusoidal gold pattern was deposited on the moving sliders (Figure 5c).

## 4. Results and Discussion

**Figure 6:**
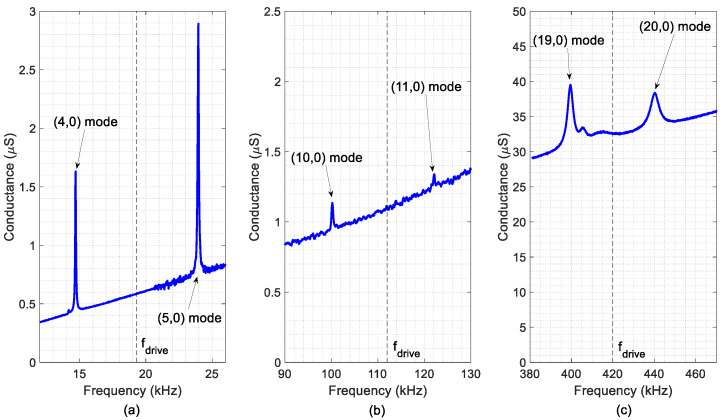
Measured conductance of three different fabricated devices: (**a**) LF, (**b**) IF and (**c**) HF. Modal identification was deduced from laser Doppler vibrometer by accounting for the number of nodal lines. The dashed vertical line indicates the driving frequency.

Figure 6 shows the real part of the admittance spectra of the different devices. Peaks corresponded to the vibration modes of interest. The modal identification was carried out with the help of the laser Doppler vibrometer. The resonant frequency of each mode and other figures of merit, such as the quality factor and the motional conductance, are summarised in Table 4. The difference in the resonance frequency between the experimental and the simulation results, indicated in Figure 2, was not higher than 5%. It is also worth pointing out that the absence of significant peaks in between the targeted modes, together with the high Q-factor and motional conductance ΔG, positively contribute to the TW generation.

**Figure 7:**
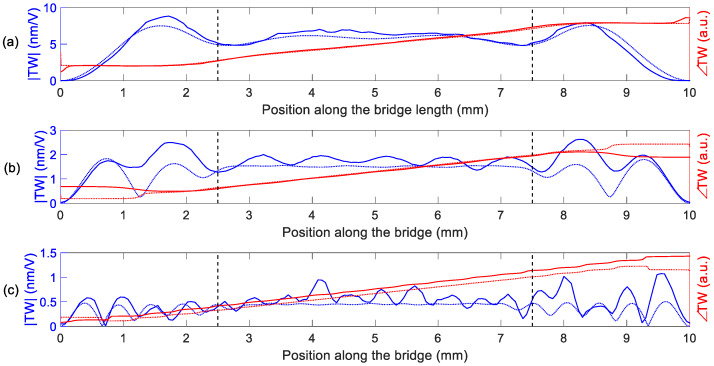
TW magnitude (per unit of applied voltage) and phase for different devices along the bridge length. Measurement (solid lines) and calculation (dotted line) in (**a**) LF, (**b**) IF and (**c**) HF devices. The dashed vertical lines indicate a centred 50% of length.

The optical characterization of the TW was performed by applying sinusoidal excitations at the mid-frequency, $f_{drive}$, (Table 4) to each of the two electrodes, with a phase difference of 90°. The measured TW are depicted in Figure 7 and the calculated SWR and <TW> are summarised in Table 5, together with the results from the simulations. As it can be seen from Figure 7, the generated TW showed low SWR values in the central plateau with a linear evolution of the phase along the bridge length, as expected. The discrepancy between the calculated and the measured SWR values was higher for the HF device, with a SWR above 3. A possible reason for this higher discrepancy may be related to the frequency spectrum of Figure 6c, where additional modes are visible between the targeted (19,0) and (20,0) resonances. These unwanted modes were not considered in the simulation model, although they clearly deteriorate the quality of the generated TW. Regarding the average amplitude of the generated TW, a good agreement between the simulation and the experiment was observed, showing a decrease for increasing frequencies. This can be attributed to the decrease in the measured Q-factor as the order of the modes increased (Table 4).

Besides their specific driving frequencies, the three devices, LF, IF and HF, were tested out of their designed frequency ranges. Results from these analyses are summarised in Table 6. As expected, the best performance in terms of <TW> and SWR required a match between the design and the corresponding driving frequency. However, at the driving frequencies of the LF and the IF ranges, TW generation was possible with all the three designs, while at the driving frequency of the HF range only the optimised device generated TW.

Looking at the results in the LF range, device LF reached the smallest SWR value while the device HF, with a larger patch (see Figure 3) and close to the one predicted by the second derivatives, generated a higher amplitude TW. This experimentally confirms the simulation results at the LF range in Table 3: the optimum patch improved the SWR at the cost of a reduction in the TW amplitude, when compared to the larger patch given by the zeros of the second derivative approach.

Once the TW generation is demonstrated and assessed, our next target was the application of the motors to transport objects in contact with the device surface. Preliminary experiments with sliders directly on top of the device surface did not lead to any reproducible result, likely associated with the poor contact due to the intrinsic bending of the bridges alongside, in the micron range. Increasing the preload, to compensate those defects, was not possible, due to the fragility of the suspended bridge structure.

To overcome this limitation, legs were attached on the device surface (see Figure 5b) to achieve a controlled and localized contact, while simultaneously amplifying the horizontal displacement at the tip of the leg. The effect of these legs on the device resonant characteristics and the TW generation was examined. The change in the resonant frequencies was below 1% and the mode shapes were unaltered. In addition, differences below 1% were observed in the <TW> and SWR of the three different designs, which corroborated the negligible perturbation caused by the implanted legs.

Next, the different experiments for the kinetic characterization of the motor–slider conveyor system are detailed. Firstly, the load capacity of the motor was tested with sliders of different masses ranging from 3 to 35 mg. Secondly, the speed of the conveyor system with a 23 mg slider was studied for actuation signals up to 8V amplitude. Finally, the positioning resolution of the system was studied, by applying a discrete number of sinusoidal cycles to the motor.

**Figure 8:**
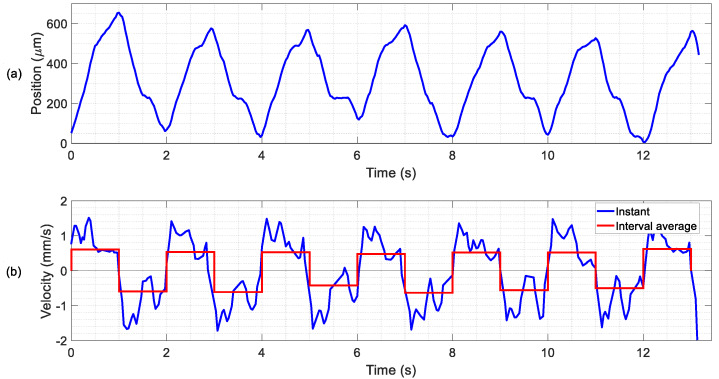
Measured results for the 3 mg slider on the LF device with 4 legs. (**a**) Slider position and (**b**) deduced velocities during the experiment with 2 V excitation signals at 19.3 kHz. The phase difference between the signals of the patches was alternated between 90° and −90° every second.

Sliders with different masses (see Table 7) were used to test the kinetic characteristics of the conveyor. In the first experiments, the lightest slider, a 3-mg silicon plate, was tested on the different legged devices. Due to the low mass of the slider, the resonant frequencies were lowered by 1% and the mode shapes remained invariant. No locomotion of the slider was detected for the IF and HF devices. This could be attributed to the lower <TW> values for the IF and HF ranges in comparison with the LF range. In what follows, only results for the LF device are presented. Figure 8 shows the experimental results with the 3 mg slider for sinusoidal actuation signals of 2 V amplitude and 19 kHz, alternating the phase difference between the two patches from 90° to −90° every second. As it can be seen in the video in Supplementary S1, the slider showed a smooth bidirectional movement with the direction determined by the phase difference between patches. Video S1 includes an inset video recorded from the microscope camera for the same experiment. With the help of the OCR software, the position of the slider could be estimated (see Figure 8a). The slider exhibited an accelerated movement in both directions of the *X*-axis, with a high repeatability. The instant velocity was calculated by numeric derivation from the position measurements, and the average speeds were also computed (see Figure 8b). The movement in both directions was clearly uniform, with an average speed of 0.6 ± 0.1 mm/s at 2 V.

**Figure 9:**
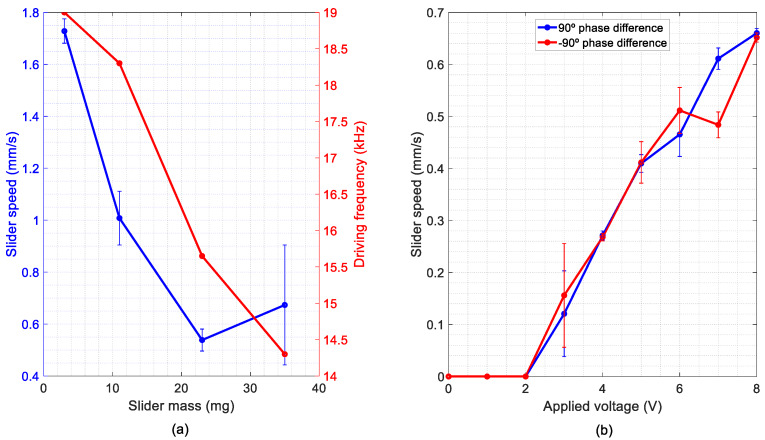
Results from the kinetic characterization of the LF device: (**a**) average speed of the sliders versus their mass for a voltage amplitude of 6 V and (**b**) average speed of the 23 mg slider at different excitation amplitudes.

Next, we focused on the maximum mass the device could transport. Voltage amplitude was limited to 6 V. Figure 9 shows the speed of the different sliders versus their mass. Our device was able to move a 500-µm-thick silicon slider with a mass of 35 mg. Considering that the mass of the vibrating bridge, with the legs, was 2 mg, the load capacity was more than 15 times the conveyor mass. The average speed decreased to 0.65 mm/s and the driving frequency had to be lowered by 23% from the unloaded case, which could be explained by the frequency shift of the modes of vibration due to the added mass. It can also be seen in the figure that the driving frequency of the TW decreased with the mass of the slider. Heavier sliders were tested, but no locomotion was obtained; an electrical impedance analysis of the devices with a 45 mg slider revealed the absence of clear resonances in the LF range. The lightest slider was able to reach an average velocity of 1.7 ± 0.2 mm/s, but due to the low mass and high speed, the contact was not stable enough, as friction force is a key factor in the motor performance. The 23 mg slider, on the contrary, showed the lowest speed, 0.55 ± 0.05 mm/s, but due to the compromise between mass and friction, this allowed a stable contact and a smooth displacement. Therefore, experiments with the 23 mg slider and different excitation voltages were carried out in order to test the speed capabilities of the conveyor (Figure 9b). The phase difference between signals was alternated every 1.5 s, so that the speed in both directions could be measured. The videos were processed following the same procedure as in Figure 8 and the average speed in both directions was estimated. As can be seen in Figure 9b, a high repeatability was obtained with low errors, and comparable behaviour in both directions. The results of the kinetic characterization showed a linear dependency of the slider speed with the applied voltage, with a sensitivity of 0.09 mm/s/V. The speed at the maximum applied voltage was 0.65 mm/s, for a slider weighting 12 times the conveyor mass. Similar-sized MEMS conveyors reported in the literature demonstrated a speed as high as 1 mm/s while carrying a factor of 11 lighter slider [28], despite being actuated by PZT, with a piezoelectric coefficient about two orders of magnitude larger than that of AlN.

**Figure 10:**
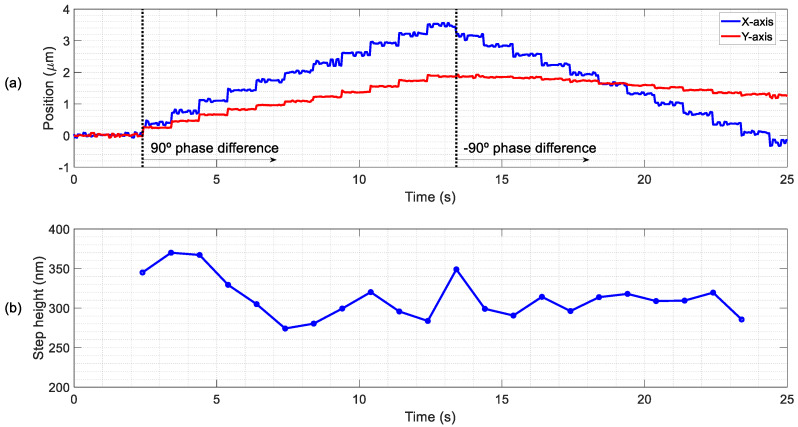
Study of the minimum displacement of the slider for 10 sinusoidal cycles of 10 V at 19 kHz. (**a**) Discrete steps on the direction of propagation of the TW (*X*-axis) and the orthogonal one (*Y*-axis) and (**b**) estimated step height.

Finally, the minimum displacements were tested and the positioning resolution of the slider was studied in open loop, without any control strategy apart from the driving signal. Instead of a continuous sinusoidal excitation, only a few cycles were applied to the patches to explore the minimal actuation that generated a TW able to move the slider between two distinguishable positions. The same experimental setup as for Figure 5c was used, in which a 3 mg slider was moved in discrete steps. The actuation for each step consisted of 10 sinusoidal cycles of 10 V at a frequency of 19 kHz, with a phase difference of 90° between patches, every 1.5 s. After 11 steps, the phase was changed to −90° to test the reversibility of the conveyor. The displacement in both the direction of propagation of the TW (*X*-axis) and the orthogonal one (*Y*-axis) were recorded. Figure 10 show the experimental results, and the corresponding video can be found as Supplementary S2. As can be seen in Figure 10a, the discrete steps were detected in both *X*- and *Y*-axis, but the *X*-axis displacement was higher, especially after the phase change. The mean step was 314 nm with a deviation of 60 nm (Figure 10b). The same experiment was carried out with a heavier slider of 23 mg and a lower applied voltage of 8 V, what allowed a minimum step value of 70 nm. This distance was close to the technical limit of the measurement setup, but even a lower minimum step could be reached by reducing the applied voltage. SAW linear actuators have demonstrated frictional driven steps as low as 2 nm, while requiring high preloads and excitation voltages, which translates to higher wear rates and power consumption [41].

## 5. Conclusions

This work reports the design, fabrication and electrical and optical characterization of piezoelectric MEMS plates for linear motion applications. The successful generation of bidirectional TW was demonstrated on a monolithic microfabricated silicon-based bridge, combined with 3D-printed legs, to overcome the intrinsic limitation of the suspended bridge to attain an efficient contact with objects.

An analytical model for the TW generation in MEMS piezoelectric plates was effectively applied to 10-mm long, 2-mm wide and 30-μm thick silicon bridges, to obtain optimum excitation patches for an efficient TW generation in three different frequency ranges: 19, 112 and 420 kHz. The experimental results were in agreement with the predicted TW figures of merit values, yielding SWR as low as 1.46 and <TW> per applied voltage as high as 6 nm/V for a MEMS bridge working at 19.3 kHz.

The kinetic performance of the fabricated motor was studied with sliders of different mass. The conveyor was able to move a 35 mg silicon slider, 18 times its weight, at 6 V and a speed of 0.65 mm/s. A lighter slider, weighting only 3 mg, was able to develop a mean speed of 1.7 mm/s at 6 V of continuous sinusoidal excitation. In addition, using a burst sinusoidal excitation comprising 10 cycles, the TW generated in the bridge surface was able to move a 23 mg slider in discrete steps of 70 nm, in both directions, which is a promising result for a TW piezoelectric conveyor of this size. 

The methodology and TW generation working principle should be applicable in lower scales, with the expected challenges: scaling down the motor area to submillimetre size would decrease the actuation of the piezoelectric film, which is proportional to the active area, but on the other hand, it would increase the working frequency of the motor.

## Figures and Tables

**Table 1:** State of the art of miniature linear motors.

Motor	Size	Actuation	Actuation	Payload	Speed	Positional Resolution	Reference
2D bidirectional	60 × 15 mm^2^	Piezoelectric	80 V	80 N	1 m/s	25 nm	[22]
1D unidirectional	10 × 10 mm^2^	Electrothermal	18 V	40 mg	8 µm/s	N.A.	[23]
1D unidirectional	15 × 5 mm^2^	Electrothermal	18 V	2.5 g	6 mm/s	N.A.	[24]
2D bidirectional	9 × 9 mm^2^	Jet air	20 kPa	2 mg	N.A.	N.A.	[25]
Rotational bidirectional	Ø3 mm	Piezoelectric	PZT, 5 V	15 mg	1500 rpm	N.A.	[27]
1D unidirectional	Array, individual size 112 × 16 µm^2^	Piezoelectric	PZT, 5 V	2 mg	1 mm/s	N.A.	[28]

**Table 2:** Electromechanical properties used in the analysis.

Parameter	Symbol	Value	Unit	Reference
Silicon density (kg/m^3^)	$\rho_{s}$	2329	kg/m^3^	[31]
Silicon Young’s modulus (GPa)	$E_{s}$	169	GPa	[32]
AlN density (kg/m^3^)	$\rho_{s}$	3260	kg/m^3^	[31]
AlN Young’s modulus (GPa)	$E_{s}$	370	GPa	[33]
AlN piezoelectric coefficient	$d_{31}$	1.28	pm/V	[30]

**Table 3:** Figures of merit deduced from the TW envelopes of Figure 4 for the two types of patches under study for the three frequencies considered. The values for the design parameters *l*_1_ and *l*_2_ are also given.

Frequency Range	LF				IF				HF			
Parameter	*l*_1_ (mm)	*l*_2_ (mm)	SWR	<TW> (nm/V)	*l*_1_ (mm)	*l*_2_ (mm)	SWR	<TW> (nm/V)	*l*_1_ (mm)	*l*_2_ (mm)	SWR	<TW> (nm/V)
Optimum patch	1.00	2.20	1.28	5.69	1.40	2.40	1.18	1.51	1.20	2.70	1.40	0.44
Zeros-based patch	0.83	3.16	1.52	8.05	0.33	1.25	1.05	1.46	0.18	0.66	1.07	0.41

**Table 4:** Device parameters calculated from the electrical impedance measurements: resonant frequency, quality factor and electrical conductance. The mid-frequency of each working range, calculated from the measured resonant frequencies is also indicated. Simulated frequencies from Figure 2 are given in parentheses.

Device Parameter	LF Range		IF Range		HF Range	
	(4,0) Mode	(5,0) Mode	(10,0) Mode	(11,0) Mode	(19,0) Mode	(20,0) Mode
Q-factor, *Q*	541	567	385	406	120	84
Motional conductance, ΔG (μS)	0.094	0.21	0.30	0.16	1.12	2.02
Resonant frequency, *f_r_* (kHz)	14.7 (13.9)	23.9 (23.0)	101 (102)	122 (125)	399 (388)	441 (431)
Mid-frequency, *f_drive_* (kHz)	19.3 (18.5)		112 (114)		420 (410)	

**Table 5:** Simulated and experimental figures of merit of the generated TW.

Device Design	LF			IF			HF		
Parameter	*f_drive_* (kHz)	SWR	<TW> (nm/V)	*f_drive_* (kHz)	SWR	<TW> (nm/V)	*f_drive_* (kHz)	SWR	<TW> (nm/V)
Simulation	18.5	1.28	5.69	114	1.18	1.51	410	1.40	0.44
Experiment	19.3	1.46	6.01	112	1.55	1.68	420	3.35	0.42

**Table 6:** Summary of the figures of merit of the different designs, each of them actuated at the three frequencies.

$f_{drive}$ **(kHz)**	19.3		112		420	
Device Design	SWR	<TW> (nm/V)	SWR	<TW> (nm/V)	SWR	<TW> (nm/V)
LF	1.46	6.01	2.5	1.64	N.A.	
IF	1.68	5.42	1.55	1.68	N.A.	
HF	1.77	7.01	3.86	1.63	3.35	0.42

**Table 7:** Sliders used in the different kinetic studies of the conveyor.

Slider (Thickness)	Length (mm)	Width (mm)	Mass (mg)
Silicon (40 µm)	10	2	3
Glass (200 µm)	11	2.4	11
Silicon (200 µm)	17.5	2.8	23
Silicon (500 µm)	12.9	2.36	35

## Supplementary Materials

The following are available online at https://www.mdpi.com/2072-666X/11/5/517/s1, Video S1: video recording of the slider speed experiment and the setup, with inset video recording from the microscope camera, Video S2: microscope video recording of the slider nanopositioning experiment.
